# Shape-specific microfabricated particles for biomedical applications: a review

**DOI:** 10.1007/s13346-022-01143-4

**Published:** 2022-03-13

**Authors:** Thomas L. Moore, Alexander B. Cook, Elena Bellotti, Roberto Palomba, Purnima Manghnani, Raffaele Spanò, Sayanti Brahmachari, Martina Di Francesco, Anna Lisa Palange, Daniele Di Mascolo, Paolo Decuzzi

**Affiliations:** Laboratory of Nanotechnology for Precision Medicine, Istituto Italiano Di Tecnologia, Via Morego, 30, 16163 Genoa, Italy

## Abstract

The storied history of controlled the release systems has evolved over time; from degradable drug-loaded sutures to monolithic zero-ordered release devices and nano-sized drug delivery formulations. Scientists have tuned the physico-chemical properties of these drug carriers to optimize their performance in biomedical/pharmaceutical applications. In particular, particle drug delivery systems at the micron size regime have been used since the 1980s. Recent advances in micro and nanofabrication techniques have enabled precise control of particle size and geometry–here we review the utility of microplates and discoidal polymeric particles for a range of pharmaceutical applications. Microplates are defined as micrometer scale polymeric local depot devices in cuboid form, while discoidal polymeric nanoconstructs are disk-shaped polymeric particles having a cross-sectional diameter in the micrometer range and a thickness in the hundreds of nanometer range. These versatile particles can be used to treat several pathologies such as cancer, inflammatory diseases and vascular diseases, by leveraging their size, shape, physical properties (e.g., stiffness), and component materials, to tune their functionality. This review highlights design and fabrication strategies for these particles, discusses their applications, and elaborates on emerging trends for their use in formulations.

**Graphical abstract:**

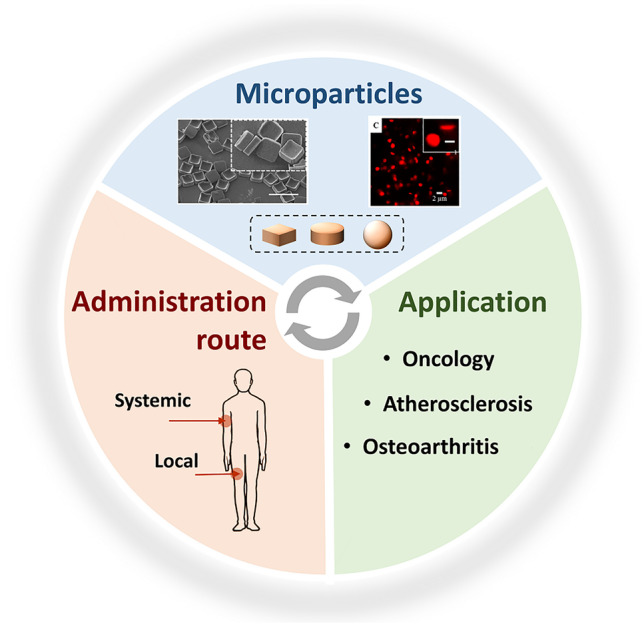

## Microplates and discoidal polymeric nanoparticles as controlled release systems

The well-known history of controlled release can be traced back to the 1950s and 1960s [1, 2], and since the turn of the millenium the focus has been on developing particle drug delivery systems on the nanoscale. However, advances in microfabrication techniques, coupled with new understandings of particle behaviors in the body, necessitate a re-examination of micron-sized drug delivery vehicles. Micron sized particles, used for either prolonged local delivery (i.e. as a drug delivery depot) or for systemic delivery, are attractive due to their capacity to load a large total amount of therapeutic, the ability to precisely tune the geometry to fit a niche application, and their versatility (i.e. being able to interchange the materials or therapeutic load). Polymers are the preferred materials for the fabrication of particles for drug delivery, due to their versatility and efficacy. In particular, poly lactic-co-glycolic acid (PLGA) and polyethylene glycol (PEG) are widely accepted in the drug delivery community because of their well-known biocompatibility, biodegradation, and excretion profiles even in humans [3–6]. Another advantage of such polymeric materials is that they can be easily modified to trigger the release of the incorporated therapeutic depending on specific physiological or external stimuli, as well as the degradation of the polymer matrix [7].

Recent advances in polymer science and fabrication techniques have opened up the possibility to engineer polymeric particles with precise control of geometry, morphology, surface properties, and functionalization. Among the others, the shape of nano- and microparticles has emerged as an important independent parameter to control drug release [8, 9]. Specifically, the shape affects the volume-to-surface ratio of the particles, thus impacting the release of the drug in the surrounding microenvironment. Moreover, the spatial organization of the polymer chains is strictly connected to the particle’s shape, leading to a different distribution and size of the pores within the matrix, with a consequent modulation of the drug release profile. This implies that new design approaches and fabrication techniques are required to properly tune the shape and geometry of polymeric particles and optimize their performances in the biomedical/pharmaceutical field.

Non-spherical nano- and microparticles have been proposed recently as an alternative system for systemic and local drug delivery to treat a variety of pathologies [10–16], and advances in nano- and microfabrication techniques have enabled the fine tuning of particle’s size and geometry with a great impact on how such particles interact with the surrounding environment, as well as on the modulation of the drug release profile.

**Fig. 1:**
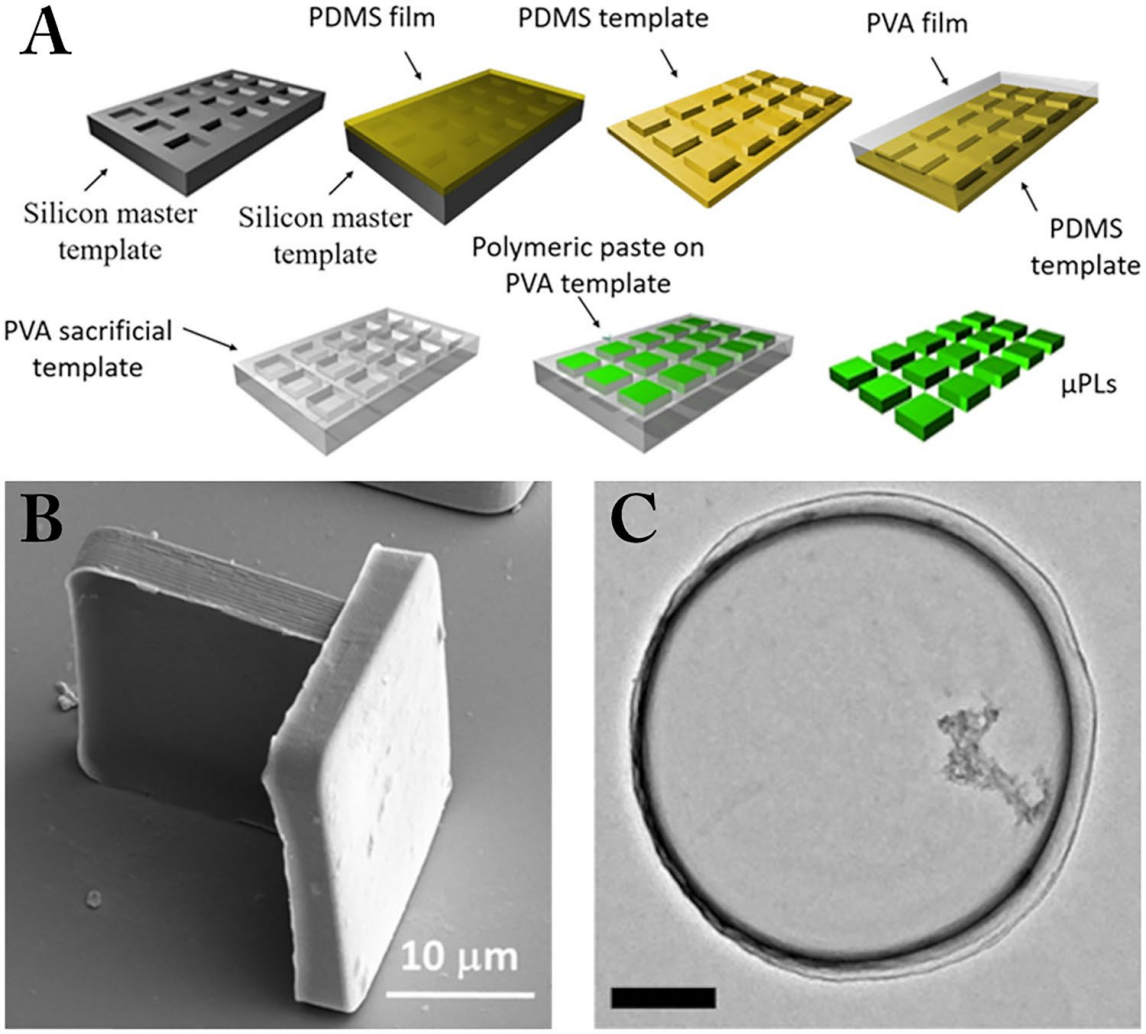
Fabrication and characterization of microplates and discoidal polymeric nanoconstructs. **A** The top-down fabrication method for both microplates (μPL) and discoidal polymeric nanoconstructs (DPNs). **B** Scanning electron microscope image of a μPL. **C** Transmission electron microscope image of a DPN. Scale bar equals 400 nm. Adapted with permission from [10]. © 2018 American Chemical Society

Here, overviews of two specific “micro” drug delivery systems are presented–microplates (µPLs) and discoidal polymeric nanoconstructs (DPNs) for a range of pharmaceutical applications. µPLs (Fig. 1B) are micron-sized, square polymeric particles that act as a local therapeutic depot [10, 11], while DPNs (Fig. 1C) are disk-shaped polymeric particles with a cross-sectional diameter in the micrometer range, a thickness in the hundreds of nanometer range, and are intended for a systemic administration [12–14]. Both kinds of particles are fabricated using a strategy that combines lithographic techniques, template molding, and polymer chemistry that allows for the simultaneous and independent control of the size, shape, surface properties, and mechanical stiffness, to tune their functionalities. The possibility to precisely tune these characteristics makes µPLs and DPN versatile particles that can be used to treat several of different pathologies such as cancer, inflammatory disease, and vascular disease. In this review, we highlight the design and fabrication strategies for these particles, discuss their applications, and elaborate on emerging trends for their use in formulations.

## Fabrication of microplates and discoidal polymeric particles

In order to tailor the physiochemical properties of particle-based drug delivery systems to the desired administration route and pathology, a variety of top-down or bottom-up methods can be employed (Table 1). Bottom-up approaches (e.g., self-assembly of amphiphiles, polymerization induced self-assembly, precipitation/crystallization, microfluidic mixing, and spray-drying techniques) can be simple to scale up and can achieve control over particle internal structure, but are beyond the scope of this review. Additional information can be found in a number of excellent recent review articles [17–19]. Top-down fabrication approaches for the preparation of microparticles with precise size and shapes have become more accessible to researchers over the past decade [20–24]. These approaches rely on the a priori definition of the physical features of such particles, conversely to more common self-assembly fabrication techniques (bottom-up approaches), whose outcome is very often spherical. The so-called top-down techniques, such as template-based soft lithography [25], particle replication in non-wetting templates [26], and continuous flow lithography [27], have multiple benefits including access to complex particle shapes, particle monodispersity, and ability to use many different polymer functionalities to form the particles. The Decuzzi group have optimized a template-based soft lithography approach to produce uniform particles of various sizes and shapes from various polymer precursors, PLGA, and PEG-diacrylate [13].

**Table 1:** Top-down and bottom-up fabrication methods for polymeric microparticles

Classification	Method/technique	Comments
Bottom-up	Emulsion/solvent evaporation	Particle formation is driven by self-assembly processes due to interaction between polymer chains and a solvent mixture
Bottom-up	Microfluidics	Similar to emulsion/solvent evaporation, particle formation is driven by self-assembly processes but within a microfluidic system that facilitates mixing
Bottom-up	Spray drying	Polymer/solvent are sprayed through an atomizing nozzle into air, which rapidly evaporates the solvent phase to form solid particles
Top-down	Grinding/milling	A coarse top-down technique, microparticles are formed by physically disrupting a polymer film or bulk material and breaking it down into smaller microscopic pieces
Top-down	Particle stretching	Polymeric particles can be cast into a sacrificial film that can then be stretched uni- or biaxially, deforming the particles into the desired shape
Top-down	3D printing	Using photopolymerization, particles can be “drawn” at high resolution using a 3D printer to get particles of complex geometries, althought this approach is time consuming
Top-down	Soft lithography	Stamping, rolling, or a mold are used to form particles at the micron and even nanometric resolution
Top-down	Continuous-flow lithography	In continuous flow lithography, particles are photopolymerized under flow with a mask that can yield controlled geometries

DPNs and μPLs are fabricated in the same general process [10, 14]. A silicon master template with microscale/nanoscale wells, in the shape of the desired particles, is formed by direct laser writing or optical lithography, combined with inductively coupled plasma-reactive ion etching, in order to have a precise top-down fabrication approach. The silicon master is then used to form an inverse polydimethylsiloxane (PDMS) replica, which is then used to recapitulate the silicon master by casting a polyvinylalcohol (PVA) film at 5–10 weight % in water onto the PDMS molds. This PVA film acts as a sacrificial template in which the desired polymer or polymer mixture (e.g., PLGA in chloroform with no crosslinking required or PLGA/PEGDA mixtures with additional photoinitiated crosslinking) can be filled. Dissolving the PVA template yields highly uniform particles of the desired geometries and sizes, and loading of therapeutics can be achieved in the same step as polymer particle formation by including drug molecules in the precursor solutions (Fig. 1).

This developed process for size- and shape-defined particles offers many advantages. One of the primary benefits is that these systems can be applied as platform technologies with interchangeable variables, including particle composition, particle size and shape, and the therapeutic compound encapsulated. This allows both the study of fundamental particle structure–activity relationships, as well as targeting of different diseases. One of the challenges of these top-down particle fabrication technologies is that scaling template-based methods to industrial levels requires considerable process development expertise or potentially redesign to fit roll-to-roll or continuous-flow techniques.

## Discoidal particles for systemic delivery

### Particles in systemic administration

The systemic (i.e., intravenous) administration of drugs is based on the transport and diffusion of a therapeutic compound inside the circulatory system, and due to the small molecular size and the porosity of the endothelium, this drug can potentially reach most parts of the body resulting in systemic side effects. As an example, the chemotherapeutic drug doxorubicin can produce cardiotoxicity [28] and bone marrow suppression [29], and many other similar chemotherapy drugs have adverse systemic effects.

The introduction of micro- and nano-particles in medicine aims to optimize treatment by improving the bioavailability and blood longevity of the selected therapeutic and by increasing its accumulation at the biological target (thus reducing side effects). Completely eliminating side effects is of course an ideal scenario that, to date, is impeded by some real-world limitations in particle drug delivery. Considering intravenous administration, which is the most common for such formulations, the main limitations are (i) the high accumulation of particles inside organs of the reticuloendothelial system (RES) (i.e., the liver, spleen, and lungs) [12] and (ii) the poor extravasation of particles, which also depends by the size of the vector itself [30]. While the former reduces the circulation time of particles, the latter makes it very difficult to reach the target tissue. RES clearance and poor extravasation are commonly accepted as the two most critical among the so-called “biological barriers,” with serum opsonins and degrading enzymes (which can favor particle clearance), and the intracellular endo-lysosomal system (tasked with degrading a certain amount of internalized objects) completing the list [31].

The accumulation of microparticles by the RES depends on the presence of resident macrophages inside the vascular lumen [12]. These cells, which are part of the immune system, continuously sense the surrounding area in order to catch and clear particulates of different origins (i.e., cell debris, pathogens). The biological origin of this process is part of regular immune-surveillance conducted by phagocytic cells, both inside organs and within the circulatory system, and represents a fundamental process in immunity [32]. Once microparticles are injected systemically, circulation will carry them past the organs of the RES and subsequently in close proximity to macrophages which may recognize these objects as potential intruders to be removed. It is important to note that the liver, spleen, and lungs are highly vascularized organs, and the presence of macrophages is considerable [33. As such, the mammalian body is programmed to eliminate particles via the RES organs and this represents a problem when designing microparticles for therapy or diagnosis.

Moreover, recent studies have demonstrated that physico-chemical particle factors and biological phenomena mediate macrophage-clearance of blood-borne particulates [34–37]. This is mainly dependent on (i) the biomolecules (including opsonins) adsorbed to the particles [38] and (ii) their stiffness [12, 39, 40]. Both parameters influenced the design of micro- and nanoparticles to decrease clearance by macrophages. PEGylation of particles, for example, is an efficient modification that reduces opsonin adsorption on the particle surface [41]. The possibility to modulate particle stiffness and design particularly soft particles is another means to minimize RES clearance of particles, and this approach was consciously chosen as a way to prolong DPN circulation time following systemic injection [12, 25]. The rationale to use a very soft particle to inhibit particle clearance by macrophages is supported by the mechanisms by which old red blood cells (RBCs) are eliminated from the blood. While aging, RBC becomes more rigid over time. Their removal from circulation is a process moderated by resident macrophages of the spleen, and only occurs when their stiffness allows phagocytic cells to correctly orient and engulf them. Flexible RBCs cannot be internalized since macrophages cannot correctly anchor their surface [42]. In line with this reasoning, recent literature provided evidence that deformable DPNs are less subject to phagocytosis in RES organs. A recent study showed how DPNs with a Young’s modulus varying over 2 orders of magnitude (from 100 kPa to 10 MPa) can be realized [12]. The same study reported that both primary rat bone marrow-derived macrophages and the conventional murine macrophage cell line RAW 246.7, preferentially internalize DPNs over a certain bending stiffness threshold, Eh^3^, equal to 3 ∼ 7 × 10^6^ k_B_T, while soft particles (below this threshold) escape more efficiently from internalization. It is important to note that, besides stiffness, also particle shape can influence the uptake. Ellipsoidal particles are in general more favorably taken up by macrophages compared to conventional circular DPNs. It is also important to underline that this shape-dependent trend is partially due to the fact that the ellipsoidal geometry confers a higher Young’s modulus and thus a higher bending stiffness [12]. In general, by using circular soft and rigid DPNs, it was proven that, after systemic injection, a minor amount of soft particles remains entrapped in liver Kupffer cells with respect to their rigid counterpart [25]. Moreover, soft DPNs accumulate to a greater extent in subcutaneous U-87 MG human glioblastoma and B16-F10 murine melanoma tumors following intravenous administration compared to rigid DPNs [25]. A possible explanation of this behavior is that soft particles are able to avoid being embraced by phagocytic cells. Deforming their structure upon contact, soft DPNs are less phagocytized and thus able to navigate the circulatory system longer and eventually depositing a larger number at the original biological target.

Due to their size in relation to “pore size” in the vascular endothelium, microparticles are poor at extravasating and thus require a rational and considered strategy to optimize delivery. This, of course, can be tailored to the specific pathology or by a series of modifications and functionalization to the particle surface. However, some general considerations need to be made. If we consider it to be impossible for a micrometric particle to extravasate, the only process which can be optimized is particle accumulation in the vasculature at the diseased site [25]. This process strictly depends on (i) particle margination and adhesion to the walls of the diseased tissue vessels and (ii) the pathology of those vessels (e.g., tumor vasculature is profoundly different from healthy vessels). It is important to note that, despite the importance of particle surface functionalization, taking into consideration particle geometry to enhance marginalization in circulatory flow can completely change the game. Thus, DPNs are designed to optimize margination, and discoids have a greater tendency to navigate the vessels (in presence of blood) at their margins compared with spherical particles.

When a particle formulation is injected into the blood flow, particle trajectory is governed by the interplay between hydrodynamic forces, near-wall lift force, and adhesive interactions of particle ligands receptors on the endothelium [35, 43, 44]. Typically, blood flow is characterized by erythrocytes migrating to the low-shear zone in the channel core by virtue of their deformability. Concomitantly, the leukocytes and platelets marginate laterally towards the vessel wall due to low deformability and their collisions with the erythrocytes. This phenomenon has inspired the design of polymeric drug delivery formulations, since increasing particle interaction with the vessel wall can promote drug transport past the endothelium into the tissue [45–49].

The size of the particle formulation determines its ability to marginate. Through mesoscopic hydrodynamic simulations, Müller et al. [50] demonstrated that microparticles perform better than sub-micron particles for drug delivery, and nanoparticles marginate poorly compared to microparticles (1–8 µm) since they require external forces to marginate effectively under hydrodynamic flow [51–53]. Microparticles exhibit higher interaction with the erythrocytes as well as tumbling motion in flow [54], and equilibrium positions of microparticles are determined by their “4S” properties (i.e., size, shape, surface functionality, and stiffness). While spherical microparticles can exhibit margination in the presence of external forces or through collisions with erythrocytes, anisotropic microparticles exhibit selective localization within micro-vessels akin to cellular components of blood [55]. For instance, the margination probability of ellipsoidal particles has been shown to be larger than that of spherical particles for equivalent particle volumes. This can be attributed to the ability of the anisotropic microparticles to undergo rotational motion in the absence of external forces [56]. Through theoretical analysis, discoidal microparticles show maximum propensity to marginate in linear laminar flow. Thompson et al. [57] have reported that the margination of microparticles differs depending on the size of the blood vessel (i.e., shear rates) as well. In the low shear rate (200 s^−1^), no obvious difference was reported among spherical and ellipsoidal particles, while under the high shear rate (500 and 1000 s^−1^), the ellipsoidal particles exhibited a higher binding rate to the vessel wall than spherical ones. Moreover, discoidal microparticles offer a large surface of adhesion and a smaller cross-section, leading to lower hydrodynamic forces and large adhesive interaction with vessel walls [35].

Microparticles with sizes greater than 3-µm pose the risk of being taken up by macrophages and could potentially occlude blood vessels. This can be addressed by tuning microparticle stiffness [43]. As erythrocytes age, they undergo a marked decrease in their deformability leading to sequestration in the spleen. Using this cue, Merkel et al. [58] studied the effect of red blood cell-mimicking hydrogel microparticle deformability on blood circulation and correlated an eightfold decrease in hydrogel microparticle elastic modulus to a 30-fold increase in the circulation half-life. The ideal formulation would then be capable of laterally drifting towards the walls to interact with the vessel endothelium but also be able to deform to avoid non-specific clearance. Moreover, the size is critical in determining the hemocompatibility of microparticles when administered parenterally. The human body naturally produces microparticles under pathologic conditions (e.g., cancer, endothelial alterations, inflammation) [59–61], resulting from membrane blebbing as part of cell apoptosis [62]. Artificial microparticles (e.g., DPNs) can mitigate this risk by minimizing protein adsorption (e.g., through their materials) and by tuning the particle materials properties (i.e., particle deformability and size). By controlling these physico-chemical properties, occlusions of narrow, distal vessels can be avoided as a particle size, surface corona, and deformability control macrophage uptake and aggregation [12, 14].

### Vascular disease (systemic administration)

Vascular diseases consist of a number of different conditions that affect central, peripheral, venous, and arterial blood flow and are due to alteration of the endothelium. The endothelium is the layer of cells that coats the inner lining of the blood and lymphatic vessels, allowing fluid, cell, and nutrient transport around the body. Moreover, it is crucial for innate and acquired immunity and for the regulation of vasomotor tone. On a molecular level, the endothelium regulates the transport of fluids and solutes between the blood and tissues. Although the endothelium is semipermeable and in the basal state, there is a continuous passage of substances through the vessel walls, and permeability can be regulated by specific external signals.

Damage to the endothelium or homeostatic dysregulation, due to and guided by inflammation and hypoxia, is observed as a result of tumors, heart attack, or other pathological conditions. Such damage often causes a deleterious increase in endothelial permeability [63]. Furthermore, endothelial damage and a pro-inflammatory environment increase the adhesion of circulating immune cells (monocytes/macrophages, neutrophils, leukocytes). With the progression of inflammation, the infiltration of immune cells through the vessel walls, along with the release of matrix metalloproteinase and other proteolytic molecules, destabilize endothelial integrity, and increasing vessel permeability. DPNs are potentially useful tools for the treatment of vascular diseases due to their ability to marginate towards the walls of the blood vessels, interact with damaged vasculature, and for their good average circulation half-life (around 24 h).

In 2018, Colasuonno et al. [14] proposed an “armed” version of DPN against ischemic stroke, a dangerous medical condition wherein the cerebral blood supply is impeded. The reduction in vascular flow due to the formation of a clot or an embolus causes a damage to the surrounding brain tissue, in that cerebral tissue, due to its high metabolic and energy needs to function properly, is highly sensitive to the lack of oxygen and glucose. The duration of the vascular occlusion, together with the location of the occlusion in the vascular network (main arterial occlusion or in microcirculation), is directly related to the severity of the damage. In the cerebral tissue, hypoxia and the lack of glucose block the fine regulation of ions and neurotransmitter trafficking in neurons. Cell depolarization and accumulation of water, ions, and neurotransmitters in the extracellular space increase brain edema, inflammation, cell excitotoxicity, and apoptosis. Thus, stroke is a difficult-to-treat condition, where the timing of treatment is crucial to reduce the negative effects of the event.

Endothelial cells, during cerebral ischemia, release plasminogen activators in the intravascular space to promote plasmin-induced lysis of clots. Plasminogen activators are also released at the same time by perivascular astrocytes located in the cell-basement membrane-astrocyte interface which affects the endothelium and increases the permeability of the blood–brain barrier (BBB) [64]. In the armed-DPN approach, DPNs are conjugated with tissue plasminogen activator (tPA), the most commonly used treatment for patients with acute ischemic stroke [14]. tPA is a serine protease that induces the conversion of plasminogen to plasmin, triggering the lysis of fibrin clots. Although tPA is a life-saving drug, it has been shown since the first clinical administration that the use of this treatment is beneficial within a short time interval of up to 4.5 h after the ischemic event [65]. Conversely, delayed administration of tPA increases the occurrence of cerebral hemorrhage and other negative side effects. It has been found that following ischemia and increased permeability of the BBB, tPA can extravasate and accumulate in the brain tissue, where it can act as a cell stimulus, worsening the condition of excitotoxicity and increasing cell death [66]. tPA-DPNs therefore have multiple advantages: the conjugation of the drug on the surface of the particles prevents the undesired spilling of the drug from the blood vessels (even under condition of increased vascular permeability), the conjugation of tPA on particles is stable (less of the 10% of the drug can detach from the particle), and lastly conjugation tPA to DPNs protects the drug from inactivation by serum proteins, thereby preserving its pharmacological activity.

It has been observed that 70% of the original activity of tPA is maintained even after 3 h of incubation of tPA-DPN in FBS [14]. The efficacy of tPA-DPN has been confirmed in vitro by thrombolysis tests under both static and dynamic experimental conditions and in vivo in the murine model of mesenteric vein thrombosis. Under static in vitro experimental conditions, the dissolution of the clots is comparable with free tPA at the same concentration. However, under dynamic conditions using a double-channel microfluidic chip to simulate clot formation and dissolution, tPA-DPN reduced the clot area by about 50% after 60 min, faster than free-tPA which took 90 min to achieve the same effect. This last result was also confirmed in the mesenteric mouse model, in which the 2.5 mg/kg concentration of tPA-DPN showed more efficacy than free-tPA to recanalize clotted vessels. In fact, 2.5 mg/kg of tPA-DPN recanalize 90% of the occluded vessels with a 50% reduction of the clot size in 35 min. Conversely, free-tPA at the same concentration recanalize 40% of the vessels, with a 20% reduction of the clot size in 35 min. All these experimental evidences can be attributed to a combination of features of the DPN as size, shape, deformability, and adhesive interactions with fibrin of the blood clot. The combination of size and shape leads to the margination of the particles near the vessel walls, favoring the interaction with the endothelium and the clot. The Young’s modulus of these particles is similar to that of cells and ranges between few a tens and a few hundreds of kPa. The softness of DPN can promote the trapping and accumulation of these particles inside the fibrin network. Moreover, the presence of tPA on the surface increases the adhesiveness of the particles to the clot due to the significant affinity between tPA and fibrin, and for the possibility of having multivalent interactions between the various fibrin molecules in the clot and the multiple tPA molecules on the particle surface. Furthermore, in the presence of more permeable BBB, the stable conjugation of tPA on DPN prevents the drug from exiting the damaged and more permeable vessels. These features, in addition to the experimental data obtained, are a good premise and show how this DPN technology is a good basis for future applications in vascular disease.

### Cancer (local and systemic administration)

Since Matsumura and Maeda [67] reported on the enhanced permeability and retention (EPR) effect for the delivery of macromolecules in cancer therapy, there has been a prevailing philosophy to design nanomedicines for cancer imaging and treatment characterized by nanoparticles with a spherical shape, an average diameter of 100 nm, and a surface mostly decorated with polyethylene glycol (PEG) chains [67, 68]. Particularly, Maeda et al. [69, 70] observed in pre-clinical in vivo models that endothelial cells of tumor vessels are not tightly connected but they rather exhibit irregular fenestrations ranging in size from several tens up to a few hundreds of nanometers. This peculiar characteristic has stimulated scientists in developing a plethora of blood-borne spherical nanoparticles sufficiently small to pass through these fenestrations and be retained within the diseased tissue. This variety of nanoparticles relies on self-assembly and colloidal interactions and differ in their material compositions, sizes, and surface properties. Specifically, both organic (e.g., lipids, polymers, block copolymers), and non-organic (e.g., iron oxide, gold, silver) materials have been employed [71–73]. Nanoparticle surfaces have been modified with different coatings, including lipids, stealth polymer chains that enhance particle blood longevity, and a variety of moieties for recognizing specific cancer cell molecules enabling what is known as active targeting [74–78].

Indeed, the US food and Drug Administration approved the first EPR-dependent nano-drug, known as Doxil, in 1995, putting in the spotlight nanotechnology and its benefits in the fight against cancer [79]. However, the universal utility of the EPR effect in the fight against cancer has recently been re-scrutinized, and alternative delivery strategies are necessary to facilitate the delivery of therapeutics to tumors [80–82]. Particularly, recent studies have shown that the EPR effects might not be as clinically relevant as it is in mice, as the size of the irregular vascular fenestrations and their density depend on the type and the stage of the tumor. These data have stimulated scientists to explore vascular targeting as a complementary therapeutic option [83, 84]. This more general vascular targeting delivery strategy is supported by another hallmark of tumor physiology, regardless of cancer type and stage: the disorganized vascular architecture leading to impairment of blood perfusion [68].

In this field, the authors have extensively demonstrated the need to finely tune nanoparticle size, shape, surface properties, and mechanical stiffness, the so-called 4S parameters, to boost tumor accumulation. Following first in silico and in vitro studies with in vivo tumor models, the authors have selected the discoidal shape and a micrometric size as the optimal parameters for nanoparticles to enhance their deposition in tumor vasculature by mitigating hemodynamic forces which can dislodge the particles.

Once in circulation, discoidal nano-constructs, exhibit the propensity to drift laterally in the “cell-free layer,” while spherical particles flow within the vessel core together with RBCs. Additionally, as compared to spheroids, discoidal particles present a larger surface suitable for interacting with the vessel walls. Regarding size, Decuzzi and collaborators demonstrated that discoids with nanometer size and thus a limited surface interaction would minimally feel the shear stress, thus accumulating in nonspecific areas. On the contrary, the interaction between the vessel walls and large discoids would be prevented by the same dislodging forces. Among the others, the mechanical stiffness has been revealed by the authors as the most critical parameter to control. Its role in controlling blood longevity, organ biodistribution, and tumor accumulation was investigated by the authors [12, 13, 85–90].

**Fig. 2:**
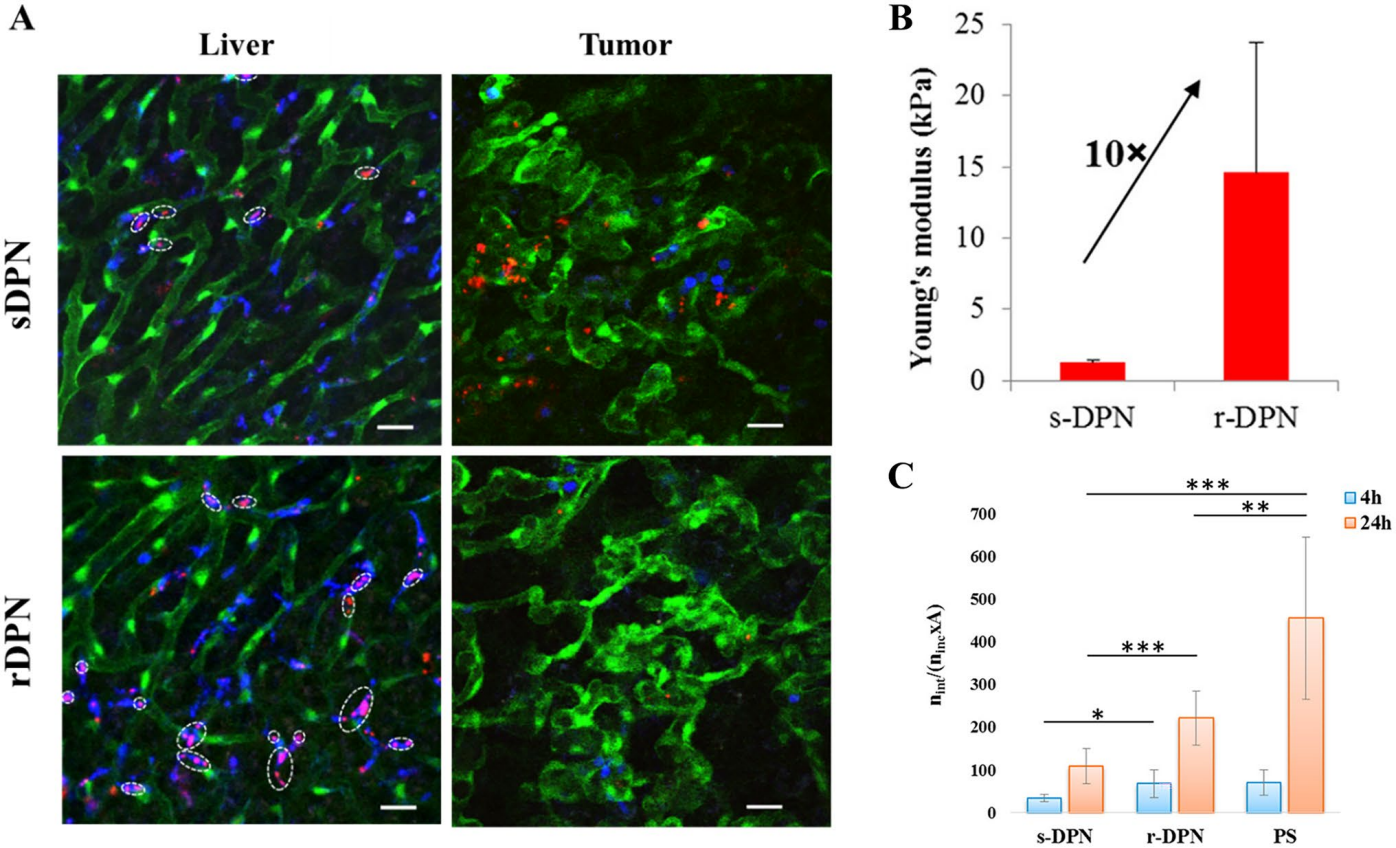
Performance of DPNs in preclinical cancer models. **A** Representative intravital microscopy images of 1-µm soft and rigid DPNs (red dots) in the liver and tumor tissue of Tie-2 mice (green fluorescence–endothelium; blue fluorescence–immune cells). Scale bars: 20 µm. **B** Young’s modulus of soft and rigid DPNs. **C** Soft and rigid DPN internalization in primary macrophages. Adapted with permission from [25]. © 2015 American Chemical Society

These principles have been investigated in pre-clinical tumor models–the in vivo performances of 1-µm DPNs with different mechanical stiffness have been studied in a preclinical model of non-orthotopic brain cancer or skin cancer [25]. To conduct these studies, U-87MG glioblastoma or B16-F10 melanoma cells were inoculated into the flank of Tie-2 mice, genetically modified to express the GFP protein in endothelial cells. This mouse model has allowed the authors to directly compare the behavior of deformable and rigid particles into the main organs of the RES system, namely the liver and spleen, and into tumor vasculature by using intravital microscopy. Specifically, once tumors reach the proper size, Rhodamine dye-labeled soft and rigid DPNs were systemically injected and different regions-of-interest (ROIs) of the liver and tumor were imaged up to 24 h post-injection to quantify particle accumulation. As depicted in Fig. 2, soft DPNs were observed to efficiently accumulate in the disorganized tumor vasculature, while they are minimally arrested by Kupffer cells of the liver. On the other hand, it is evident that rigid particles co-localize with Kupffer cells, implying their rapid uptake by phagocytic cells, while their deposition into tumor vasculature was not significant. The more efficient evasion of soft DPN from immune cell recognition has also been confirmed by in vitro studies done on primary macrophages. These data would suggest that the deformability associated with soft DPNs is the main reason for the enhanced tumor accumulation, given the ability to escape from macrophage recognition and phagocytosis. Such a phenomenon should then prolong their circulation in the bloodstream, thus boosting the chances to accumulate into the disease area.

**Fig. 3:**
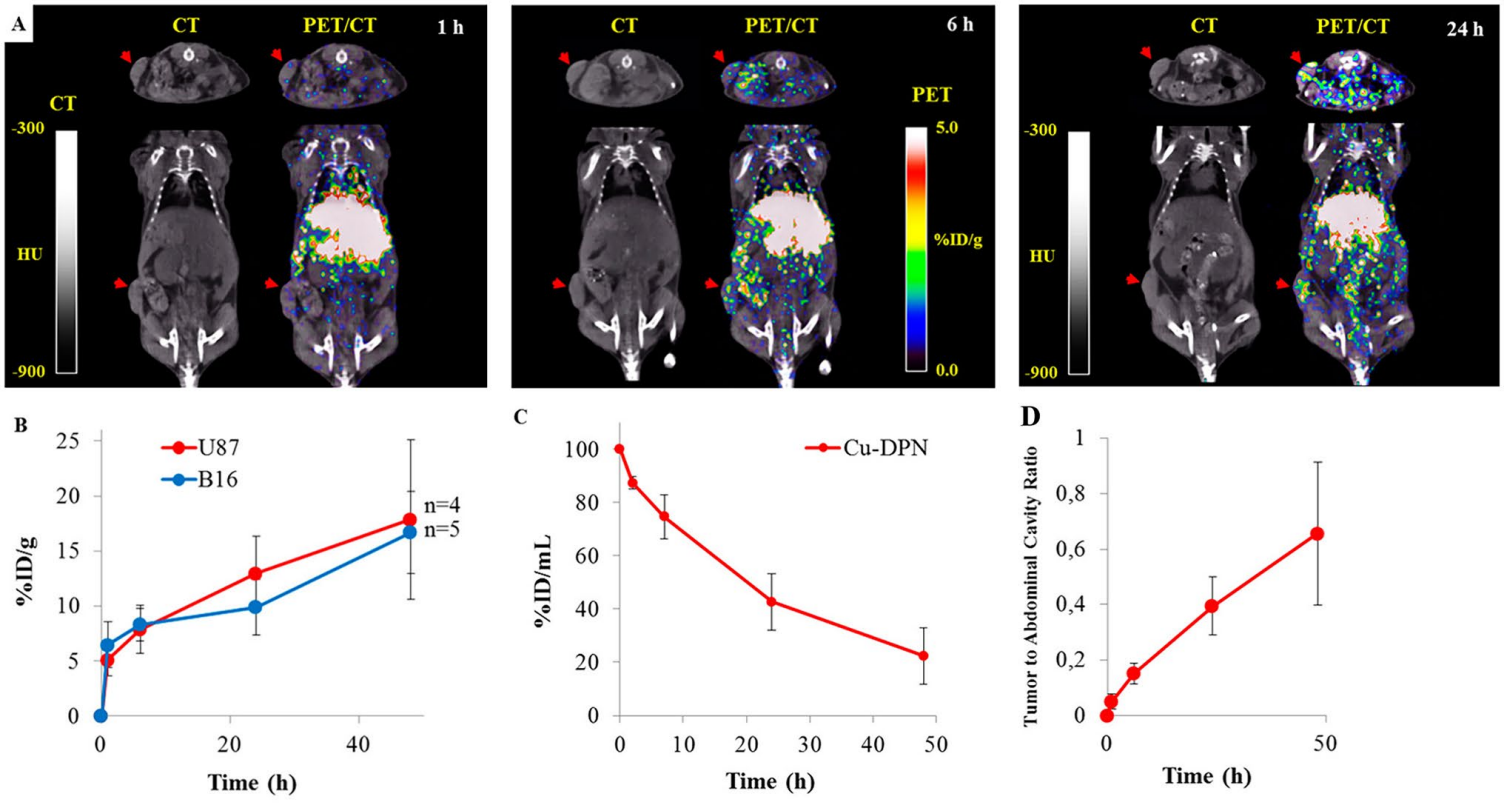
Performance of DPNs in preclinical cancer models. **A** PET/CT imaging of 1-μm soft DPN accumulation in flank implanted brain tumors. **B** Quantification of DPN accumulation in U87-MG and B16-F10 tumor bearing mice, expressed in terms of percentage injected dose per gram tissue (%ID/g). **C** DPN concentration in the blood measured via scintillation counter, at different time points (0, 2, 7, 24, 48 h p.i.). **D** Tumor to abdominal cavity activity ratio over time. Adapted with permission from [25]. © 2015 American Chemical Society

This was further demonstrated by nuclear imaging (PET/CT) of a non-orthotopic brain tumor mouse model [25]. DPNs (1-µm diameter), as soft as cells (~ 1 kPa), were able to circulate for a long period of time, showing a circulation half-life of about 20 h. As already suggested by the optical imaging studies, the long circulation of soft DPNs guaranteed an unprecedented tumor accumulation at doses equal to 20% of the injected dose per gram tissue and a low accumulation into the RES organs (Fig. 3). Overall, the authors showed that the rational design of non-spherical nanoconstructs, by finely tuning the geometry and the mechanical properties, is a fundamental step for optimizing the nanoconstruct performances in vivo, as compared to conventional bloodborne nanoparticles.

**Fig. 4:**
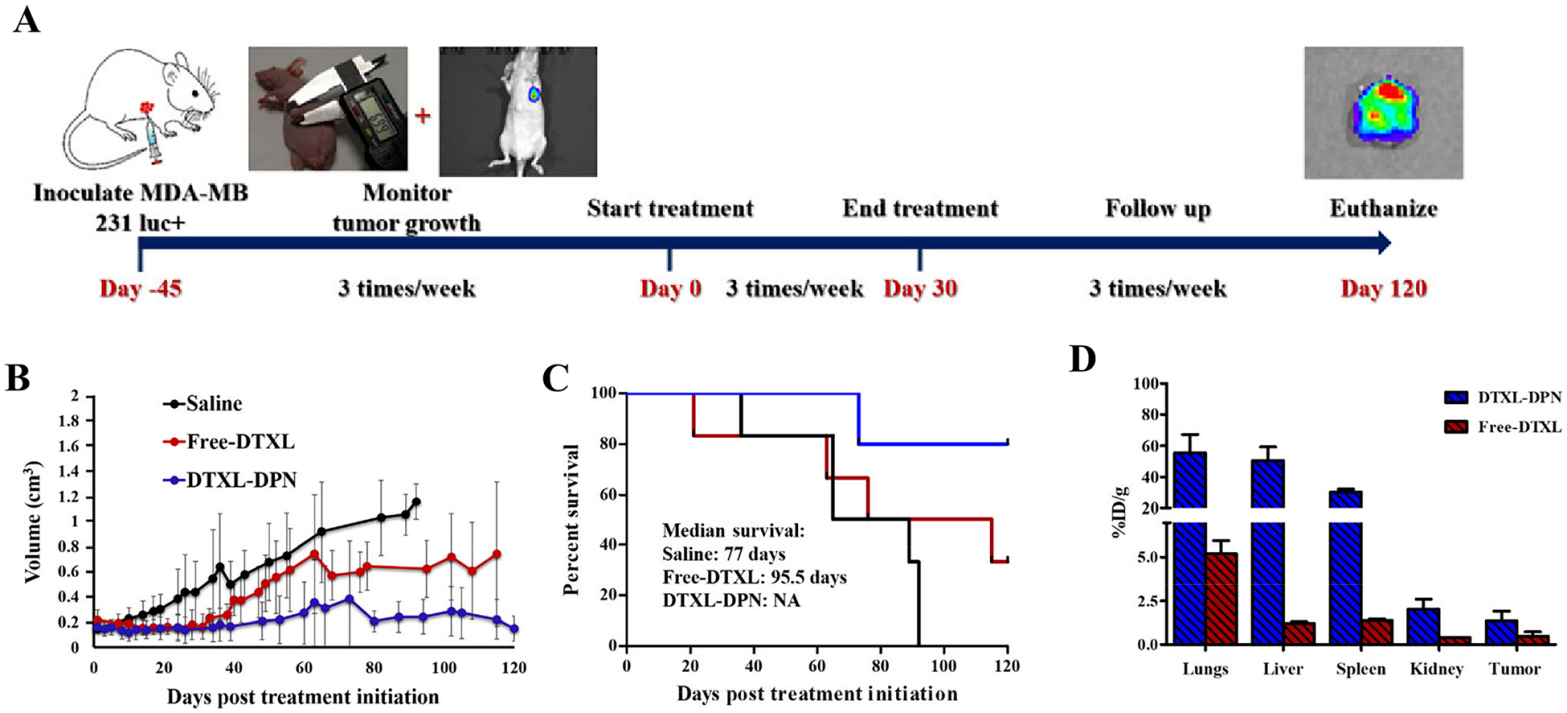
In vivo therapeutic and imaging studies on orthotopic breast cancer murine models. **A** Timeline of the preclinical experiments performed on mice bearing orthotopic breast cancer and including bioluminescence/fluorescent imaging and tumor growth analysis. **B** Average tumor growth curves comparing the efficacy of three different intervention. Data are presented as the average tumor volumes ± SD. (black line: saline; red line: Free DTXL; blue line: DTXL-DPN). **C** Kaplan–Meier curves for survival (black line: saline; red line: free DTXL; blue line: DTXL-DPN). **D** Docetaxel accumulation in major organs expressed as the percentage of the injected dose normalized by the organ mass (%ID/g) at 24 h post administration of free-DTXL (red bar) and DTXL-DPNs (blue bar) (2 mg/kg of DTXL). Adapted with permission from [91]. © 2021 Springer Nature

In a more recent work, Felici et al. [91] developed a class of DPNs to boost drug loading and release at the target site, thus improving their pharmacological and imaging properties. Specifically, 1 µm DPNs were loaded with the known chemotherapeutic docetaxel (DTXL), and the therapeutic efficacy of DPNs was tested pre-clinically in mice bearing orthotopic triple-negative breast cancer. To induce triple-negative breast cancer, mice were inoculated with MDA-MB231 luciferin positive cells, and tumor growth was followed by IVIS optical imaging and caliper measurements. Once tumors reached a volume of about 0.15 cm^3^, mice were randomly divided into groups for injection: the “saline” group (mice injected with PBS), the “free-DTXL” group (mice injected with a DTXL solution), and the “DTXL-DPN” group (mice treated with 2 mg/kg of DTXL-loaded within the DPN). In all cases, the administration was performed intravenously every 2 days for up to 30 days, at a very low dose of 2 mg DTXL/kg. Figure 4 reports the tumor growth curves for the three different treatment groups over a period of 120 days. The tumor growth plots clearly demonstrate the therapeutic advantage in using DPNs as compared to the free formulation. Tumor mass in the saline group grows continuously, and no mice survive 90 days from the onset of the study. Mice treated with the free DTXL and DTXL-DPNs initially behave similarly, with both groups showing a positive response and a stabilization of the tumor mass. However, while the initial positive outcome for the free DTXL group is followed by a progressive tumor relapse, mice treated with DTXL-DPNs respond positively for the whole observation period, showing even a reduction of the tumor mass, and most importantly no relapse of the disease. Indeed, 80% of the mice treated with DTXL-DPNs survived at 120 days, against only the 30% of the mice of the free DTXL group. To further prove the enhanced therapeutic benefit of the DPNs over the free drug administrations, the amount of DTXL into the organs was studied 24 h post a single injection of particles by using liquid chromatography–mass spectrometry (LC–MS). Data in Fig. 4D show that the amount of DTXL deposited within the tumor mass following a single DTXL-DPN injection (1.4 ± 0.6%ID/g) is about 3 times higher than that measured for the free-DTXL (0.5 ± 0.2%ID/g). This would explain the enhanced therapeutic efficacy observed for the DTXL DPNs over the free drug. These results continue to support the idea that non-spherical particles, such as DPNs, might be employed to efficiently treat a variety of malignancies independent of the EPR effect and at minimal injected drug doses.

### Microparticles for pulmonary delivery

Pulmonary delivery of microparticles is an attractive approach due to either the desire to target lung pathologies or the ability to avoid first-pass hepatic clearance and enable rapid onset of pharmaco-logical activity [92]. For aerosol/powder delivery to the alveoli (i.e., deep lung tissue), microparticle formulations must have an aerodynamic diameter < 5 μm and the optimal aerodynamic diameter is between 1 and 3 μm [93, 94]. However, microparticle-based inhalation therapies have struggled to enter the marketplace. The most well-known example is the withdrawal of Nektar Therapeutic’s and partner Pfizer’s Exubera, an inhaled formulation of insulin particles (1–5 μm) [95–97]. The rollout of Exubera was confounded by several complications, chief among these are reports by the FDA of an increased risk of lung cancer in former smokers. This news, coupled with advances in conventional “needle-based “ insulin delivery and poor initial sales, lead Pfizer to abruptly withdraw Exubera from the marketplace. Further attempts at delivering microparticle-borne therapeutics via inhalation to the lungs have since suffered from these setbacks [97].

Besides the inhalation route, it is also possible to deliver microparticles to the lungs via systemic circulation exploiting their specific size and behavior. For instance, mesoporous silicon microparticles have been shown to significantly distribute to the lungs when compared to other geometries (e.g., spherical, hemi-spherical, and cylindrical) following intravenous administration [88]. Silica spheres or silicon hemispherical, discoidal, or cylindrical particles were administered into nu/nu nude mice with subcutaneous MDA-MB-231 human breast cancer xenografts. Hemispherical, discoidal, and cylindrical particles had similar volumes (0.6, 0.6, and 0.8 μm^3^, respectively), and it was shown that discoidal particles accumulated 4 times more in the lungs compared to spherical particles and 8 times more compared to hemispherical and cylindrical particles. These data are explained by the tendency for discoidal particles to drift closer and adhere to the vessel walls and, due to their shape, minimize uptake by cells of the RES system.

The group of Key has further explored the ability of DPNs to preferentially target the lungs following intravenous administration [98, 99]. DPNs with a diameter of approximately 3 μm and a height of 1.5 μm were radiolabeled with ^89^Zr and were administered intravenously to healthy Balb/c mice via tail-vein injection [98]. There was significant accumulation of DPNs in the lungs at 2 h after injection, as measured by PET/CT imaging. The level of DPNs in the lungs remained high 1 day after injection and was detectable up to 7 days after injection. In a follow-up study, Park et al. [99] investigated DPNs (nominally 3 μm in diameter and 1.5 μm in height) loaded with curcumin for the treatment of an asthmatic mouse model. The preclinical model was established by sensitizing mice with an intraperitoneal injection of ovalbumin, and then, DPNs loaded with curcumin and the fluorophore cyanine 7 were injected intravenously. Ex vivo fluorescent imaging of organs following injection showed an initial accumulation of DPNs in the lungs, and the therapeutic efficacy of curcumin-loaded DPNs was observed by the reduction of inflammatory cells obtained via bronchioalveolar lavage fluid analysis and reduction in bronchial wall thickness. These two recent studies indicate that, rather than administering microparticle formulations via inhalation, the lungs can be targeted via the systemic administration of particles where the specific shape can enhance pulmonary deposition.

## Microparticles for local delivery

Since the 1970s, extensive research has been conducted in order to develop more efficient ways of delivering therapeutics [73, 100–103]. Oral administration offers several advantages and is one of the most widely used route of administration. However, it is severely limited by low bioavailability and is not optimal for a wide range of therapeutics, including proteins and peptides [103, 104]. In contrast, parenteral administration routes including intramuscular, subcutaneous, and implant devices offer possible routes to circumvent these problems. The main challenge associated with parenteral administration is its invasiveness, which becomes magnified in the case of chronic disease, especially in children, since it often requires repeated dosing over longer time periods. In order to overcome these problems, several alternative administrative routes have been proposed for different pathologies like the pulmonary [94], nasal [105], transdermal [106], and ocular routes [4]. In addition to these administration routes, the use of sustained drug delivery platforms has emerged as the most promising way to overcome long periods of repeated dosing [107]. To this end, monolithic release devices with controlled or on-demand triggered release of therapeutics, through injectable or implantable devices, have emerged as a solution that offers prolonged release over an extended time periods (thereby reducing the number of administrations), potential to achieve high local drug concentration in target regions (reducing systemic exposure of the drug to non-target tissues), and the use of a biocompatible and biodegradable polymer matrices which act as a biodegradable depot [1, 102, 103].

### Use of monolithic devices in the clinic

Injectable microparticle-based depots are one of the first and, to date, one of the most widely used controlled release systems. Due to the historical significance of monolithic drug delivery depots, there are numerous examples of this type of drug delivery system in the clinic. For example, Lupron Depot^®^ is one of the first commercially marketed peptide containing microparticle-based formulation (PLGA microspheres approximately 10 µm in diameter) developed for the slow release of a short peptide hormone, leuprolide [108–110]. It was initially approved for daily administration for the treatment of prostate cancer, endometriosis, and central precocious puberty, and in 1989, an injectable, slow-releasing formulation reduced the daily administration to once every month by intramuscular injection or once in 6 months by subcutaneous injection [111, 112]. The reduced frequency of administration led to commercial success, resulting in the current annual sales of nearly 1 billion USD. Despite this success, it is worth mentioning that methods for encapsulating peptides and proteins, while maintaining their secondary structure, as well as loading similar macromolecules, remains a challenge for these microparticles [113].

Risperidal Consta^®^ is another an example of a PLGA-based microparticle platform (25–200-µm diameter) developed by Alkermes for the slow release of the antipsychotic drug Risperidone [114]. It was approved for commercial use in 2003 for the treatment of schizophrenia and later for bipolar disorder. It was typically designed using PLGA (lactide/glycolide 75:25) and administered once every 2 weeks by intra-muscular injections, but the treatment is also dependent on the oral administration of Risperidone in the first 3 weeks of the treatment [115]. This is a classic example where the drug release kinetics depends on the degradation of the PLGA microparticles. In order to overcome the problem of oral administration in the initial days of the therapy, a lactide/glycolide 50:50 PLGA copolymer has been recently developed that reported a zero-order release of Risperidone in the first 20 days [116]. There exist several other microparticle-based controlled release systems in the clinic (Table 2), and their continued use points toward the necessity to continue investigation on this class of systems.

**Table 2:** Marketed microparticles for different medical indications

Registered name/manufacturer	Drug	Particle (material/size)	Indication	Route of administration	Frequency
Bydureon®/AstraZeneca [119]	Exenatide	PLGA 75:25 1–180 µm	Type-2 diabetes mellitus	Subcutaneous	1 every week
Zilretta®/Flexion [120]	Triamcinolone acetonide	PLGA 75:25 20–100 µm	Pain killer	Intra-articular	1 every 12 weeks
Arestin®/OraPharma [121]	Minocycline hydrochloride	PLGA 28–40 µm	Gum infection	Subgingival	1 every 12 weeks
Sandostatin® LAR/Novartis [117]	Octreotide acetate	PLGA 55:45 1–250 µm	Acromegaly, carcinoid tumors	Intramuscular	1 every 4 weeks
Trelstar® LA/Debiopharm [117]	Triptorelin pamoate	PLGA ≤ 200 µm	Prostate cancer	Intramuscular	1 every 4–26 weeks
Somatropin biopartners®/Biopartners and LG Life Sciences [118]	Somatropin	Sodium hyaluronate 1–50 µm	GH deficiency, Turner syndrome	Subcutaneous	1 every week

### Drug loading and release from μPL

The success of microparticle-based delivery systems inspires further development. However, to date, most of these sustained delivery systems have been limited to a spherical particle geometry. Depending on the fabrication process, it is possible that non-spherical particles could alter drug delivery (e.g., the fabrication method changing particle properties to tune release kinetics), mediate the biological response, and even impart some therapeutic benefit. In this vein, a monolithic PLGA-based slow-release platform called µPLs have been developed for the sustained release of therapeutics [10, 11]. These are microparticle depots fabricated using a top-down method and are typically square-shaped with an edge length of 20 microns. The square base is usually 20 by 20 µm, while the height of the particles can be tuned between 5 and 20 µm. The steps of the fabrication method are similar to those for preparing the DPNs. It is important to highlight that the micrometric size ensures that they can be directly deposited at disease sites by way of a simple injection. Moreover, this size also ensures that the particles properly interact with the surrounding biological environment without being internalized by cells.

The loading of therapeutic small molecules into microparticles is often challenging. Loading the therapeutic during microparticle fabrication often leads to denaturation or destruction of the actual therapeutic component, especially in the case of proteins and RNA. Moreover, the overall polymer-to-drug mass ratio is extremely important, and to date, the excipients in the formulation are > 50% of the actual drug content. Commonly, the therapeutic molecules can be introduced (i) in their bare molecular form, (ii) in the form of therapeutic-loaded nanoparticles, or (iii) as solid dispersions (i.e., crystals). In the case of the developed µPLs, we have successfully achieved molecular loading of anti-inflammatory drugs like dexamethasone (DEX) and curcumin [11, 122]. Moreover, the fabrication method simultaneously enabled us to develop a hierarchical system where the DEX was incorporated first into a nanoparticle and then introduced into the larger microparticle.

Additionally, the fabrication method allows precise tuning of the polymer content in the particles, thereby enabling control of the particle mechanical properties as well as the therapeutic release profile.

The drug release kinetics from polymeric matrices is primarily determined by (i) polymer swelling, degradation, and erosion and (ii) the diffusion of the drug molecule through the polymer matrix (which is a function of the partition coefficient of the drug within the polymer versus water). In general, the release of the drugs takes place in two distinct phases–an initial burst release followed by a prolonged and sustained release. When drugs are loaded in their molecular form, the release is governed more by the intrinsic properties of the drug. It was possible to study the release kinetics of DEX from µPLs using two different kinds of loading. When the drug was loaded in its molecular form, 60% of the drug was released in the first 24 h while the remaining drug was released slowly over 10 days. However, when employing the hierarchical system (i.e., loading DEX into smaller PLGA particles which are subsequently loaded into µPLs), the release was further slowed and only 35% of the drug was released in the first 8 h. Another in-depth investigation was carried out to understand the effect of polymer concentration, shape, and surface area on the loading and release kinetics of curcumin and DEX. Greater amounts of PLGA resulted in more accurately shaped particles, while less PLGA resulted in more defect-prone particles. The amount of drug loaded into the particles was found to be directly proportional to the amount of the polymer present in the particles, i.e., taller and denser particles were loaded with higher amount of curcumin. The loading efficiency of DEX and curcumin also was directly proportional to the hydrophobicity of the drug molecules. Notably, higher polymer content corresponded to a slower release rate and reduced the initial burst release. This observation can be attributed to the more compact polymeric matrix of the particle. In summary, a systematic study of µPLs loading and release of multiple therapeutics highlights the potential of this delivery platform with controlled geometry. The particle size and shape can be further explored as a means for imparting a therapeutic benefit (beyond the drug delivery aspect) in the treatment of diseases, namely osteoarthritis.

### Osteoarthritis (local administration)

Osteoarthritis (OA) is a chronic inflammatory disease that affects populations of different age groups. While the common form is more prevalent in the elderly population, younger people are more vulnerable to post-traumatic osteoarthritis (PTOA) [123–125]. Due to the lack of in-depth knowledge of the disease’s progression and challenges in early diagnosis, the global impact of OA continues to increase with the increase of obesity, age, nutrition, and stress among the population [126]. Currently, all available pharmacological strategies are palliative and do not prevent, arrest, or even restrain the disease. They essentially act on reducing pain and inflammation, thus improving joint mobility. These interventions are usually combined with other non-pharmacological approaches, like exercise, weight loss, or lifestyle changes. As the first line-therapy, small molecules, such as acetaminophen (paracetamol), non-steroidal anti-inflammatory drugs (NSAIDs), opioid analgesics, or COX-2 inhibitors are the most used [127–129]. Due to the localized nature of the disease, intra-articular injection has emerged as one of the primary administration routes for treatment. The benefits of intra-articular injection include (i) direct injection of the drug in the target site, especially in populations that have existing co-morbidities; (ii) avoiding side effects connected with systemic administration; and (iii) increasing drug concentration at the target site, thereby reducing the number of administrations [130, 131]. However, this administration route is invasive and requires trained personnel; thus, it is challenging to maintain the therapeutic drug concentration over a long period of time.

For this reason, several strategies have been developed and marketed. The most common approach involves the use of monolithic polymeric depots in order to have a prolonged and sustained drug release [132, 133]. In 2017, Zirletta, PLGA microparticles approximately 45 µm in diameter, was clinically approved for extended-release of triamcinolone acetonide for the intra-articular treatment of the OA knees. This formulation was developed to increase drug residence time, promoting the continuous and sustained release of the corticosteroid at the target site over a period of 12 weeks and decreasing its systemic side effects. These polymeric particles were synthesized by solid-in-oil-in-water emulsion technique and are characterized by a size range between 20 and 100 µm. Triamcinolone acetonide is typically loaded in these particles in the form of nano and micro-crystals [134].

Based on this idea, PLGA-based µPLs were recently utilized to achieve the slow release of another clinically approved corticosteroid, DEX, for the intra-articular treatment of PTOA in a mouse model. The rationale for the use of these particles stems from their tunable mechanical properties. Di Francesco and coworkers [10] developed µPLs with an apparent modulus of 3.1 ± 0.9 MPa, a value similar to the typical values reported for the healthy cartilaginous tissue [135]. In order to establish the possible application of this platform for intra-articular OA treatment, the mechanical properties of particles were studied using nanoindentation and dynamic mechanical analysis. DEX was efficiently loaded inside the μPLs, and its release profile was studied in infinite and confined environments. Additionally, the in vitro ability of DEX-loaded μPLs to reduce inflammation was investigated in lipopolysaccharides (LPS)-stimulated ATDC5 murine chondrogenic teratocarcinoma cell line. After demonstrating the ability of these particles to be retained in the knee of the PTOA mouse model using intravital and confocal microscopy, the therapeutic efficacy of DEX-loaded μPLs was assessed in the same model. The authors demonstrated that these particles are characterized by an effective dissipation parameter tan δ of 0.3, typical of materials with high damping and shock absorbing properties [136, 137]. Thus, injection of µPLs in OA intra-articular space the has potential to ensure mechanical support of the joint, to minimize wear, cartilage laceration, and improper bone remodeling. At the same time, particles provide a continuous release over a period of 10 days at infinite sink conditions and at least 1 month in a confined microenvironment.

**Fig. 5:**
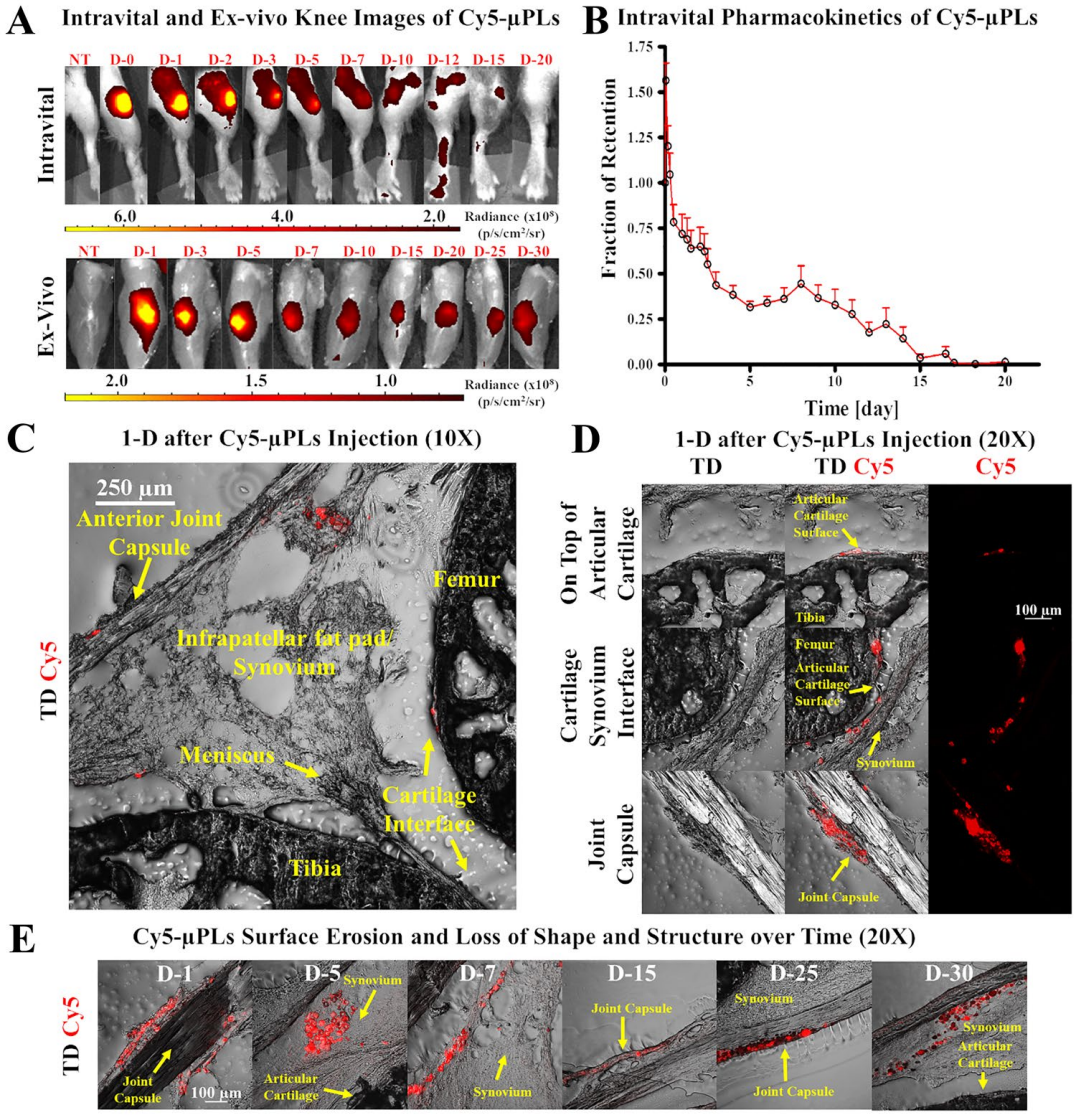
In vivo pharmacokinetic study of Cy5-conjugated microPlates (Cy5–μPLs) in a PTOA mouse model. **A** Representative pharmacokinetic time course intravital images (skin on) and ex-vivo knee images (skin off) of Cy5-μPLs injected intraarticularly into PTOA mouse knee joints (D-#, where # represents days after intraarticular injection). **B** Intravital fraction of retention of Cy5-μPLs plotted as mean + SEM. Note = the initial uptick in fluorescence within the joints in the first couple of hours after injection is a result of loss of fluorophore self-quenching, which occurs due to high density fluorophore conjugation onto the particles. **C** Anatomically labeled sagittal section of a mouse knee joint 1 day after intraarticular injection showing the Cy5-μPLs dispersed across the joint interacting and/or in close proximity to many different tissue types such as the cartilage, the infrapatellar fat pad and synovium, and the joint capsule. **D** Confocal microscopy imaging performed 1 day after intraarticular injection showing Cy5-μPLs located on top of the cartilage surface, near the cartilage/synovium interface, and the joint capsule. In all images, the scale bar = 100 μm. **E** Confocal microscopy imaging of Cy5-μPLs within the mouse knee joint taken at different time points after intraarticular injection. TD transmission detector. NT no treatment. For intravital imaging analysis, *n* = 4–24 limbs depending on the time point, i.e., earlier time points had more animals included, and the sample size at the later timepoints was lower because some animals were taken down at earlier timepoints for ex vivo and confocal microscopy analysis. For ex vivo imaging analysis and confocal microscopy analysis, *n* = 2–4 limbs per time point were well detected in the knee for over 15 days and the deposition of μPLs was seen. Adapted with permission from [122]. © 2021 American Chemical Society

The biodistribution of these particles and retention after a single intra-articular administration was studied by covalently conjugating Cy5 on the particles surface [122]. The study demonstrated that Cy5-µPLs were retained in the knee over a period 30 days (in vivo and ex vivo analysis, Fig. 5A, B) and their deposition was observed by confocal microscopy for up to 30 days in the cartilage surfaces, infrapattelar fat pad/synovium, and joint capsule (Fig. 5C, E). It was demonstrated that a single intra-articular injection of DEX-µPLs (1 mg/kg) reduced the expression of pro-inflammatory cytokines, such as IL-1β, TNF-α, IL-6, and MMP-13 and cartilage degradation. In particular, DEX-µPLs showed a better anti-inflammatory activity and an improvement of articular cartilage and synovial tissues load-induced histological changes compared to free DEX, 1 month post-injection. Interestingly, in addition, empty-µPLs reduced the expression of MMP13 compared to free DEX. This could be attributed to the mechanical properties associated with the µPLs.

## Conclusions

Micron-sized, PLGA-based drug delivery vehicles have been used clinically since the 1970s, and their market durability is a testament to the benefits they bring to the field of controlled drug delivery. While historically it has been more feasible to fabricate spherical microparticles, advances in micro-fabrication techniques enable the development of microparticles with customized and high-precision size and shape. Here, two such formulations have been examined: DPNs and µPLs.

DPNs, due to their biomimetic shape, are ideal for systemic administration–the discoidal shape facilitates margination within the vessels and can be used to deliver drugs for vascular diseases and cancer. In vascular disease, for example, DPNs are able to align in flow and marginate to the vessel periphery. Furthermore, DPNs can non-specifically target the vasculature due to their high aspect ratio nature which presents greater potential surface area to contact the vessel walls. In the treatment of cancer, DPNs are able to passively accumulate in tumor vasculature but are able to avoid clearance by macrophages of the reticulo-endothelial system due to their shape and flexibility. µPLs, on the other hand, are able to lend a physicality to the treatment of osteoarthritis (e.g., µPLs act as both a drug delivery depot and as a “cushion” in the intra-articular space). Thus, these microparticle formulations can be designed to take advantage of their geometry, size, and rigidity to enhance the therapeutic effect, while also utilizing conventional controlled drug delivery (i.e., release of a therapeutic from the polymer matrix).

The top-down fabrication approach for DPNs and µPLs offers potential for technological platforms where the particle size, shape/geometry, rigidity, material, and therapeutic payload can be interchanged and tailored to meet the physiological need. This includes the control of DPN aspect ratio, altering the polymer matrix (e.g., using hydrogels or stimuli-responsive materials), and the development of hierarchical delivery platforms. Indeed, it was already shown that the loading of drug-loaded polymeric nanoparticles within µPLs was able to modify drug release kinetics and employing such a hierarchical system could also potentially enable the co-delivery of therapeutics. Despite the top-down fabrication having a lower yield compared to particles obtained with bottom-up techniques, the progress obtained in recent years in the industrial processes has paved the way to other micro-particles made out with similar techniques to be scaled up for human clinical trials. Thus, taking together the promising advantages demonstrated by such platforms and the possibility to overcome production challenges, DPN and µPLs, with their complex and precise size and shapes, has the potential to build towards the next generation in a controlled delivery.
