# Correction: Shape-specific microfabricated particles for biomedical applications: a review

**DOI:** 10.1007/s13346-022-01180-z

**Published:** 2022-05-30

**Authors:** Thomas L. Moore, Alexander B. Cook, Elena Bellotti, Roberto Palomba, Purnima Manghnani, Raffaele Spanò, Sayanti Brahmachari, Martina Di Francesco, Anna Lisa Palange, Daniele Di Mascolo, Paolo Decuzzi

**Affiliations:** Laboratory of Nanotechnology for Precision Medicine, Istituto Italiano Di Tecnologia, Via Morego, 30, 16163 Genoa, Italy

## Correction to: Drug Delivery and Translational Research https://doi.org/10.1007/s13346-022-01143-4

Elena Bellotti's family name is correct as reflected here.

The original article was corrected.

